# Dynamic change of COVID-19 lung infection evaluated using co-registration of serial chest CT images

**DOI:** 10.3389/fpubh.2022.915615

**Published:** 2022-08-12

**Authors:** Xiao Chen, Yang Zhang, Guoquan Cao, Jiahuan Zhou, Ya Lin, Boyang Chen, Ke Nie, Gangze Fu, Min-Ying Su, Meihao Wang

**Affiliations:** ^1^Department of Radiology, Key Laboratory of Intelligent Medical Imaging of Wenzhou, First Affiliated Hospital of Wenzhou Medical University, Wenzhou, China; ^2^Department of Radiation Oncology, Rutgers-Cancer Institute of New Jersey, Robert Wood Johnson Medical School, New Brunswick, NJ, United States; ^3^Department of Radiological Sciences, University of California, Irvine, CA, United States; ^4^Department of Radiology, Yuyao Hospital of Traditional Chinese Medicine, Ningbo, China; ^5^The People's Hospital of Cangnan, Wenzhou, China; ^6^Ningbo No. 6 Hospital, Ningbo, China; ^7^Department of Medical Imaging and Radiological Sciences, Kaohsiung Medical University, Kaohsiung, Taiwan

**Keywords:** COVID-19, computed tomography, dynamic changes, registration, pneumonia

## Abstract

**Purpose:**

To evaluate the volumetric change of COVID-19 lesions in the lung of patients receiving serial CT imaging for monitoring the evolution of the disease and the response to treatment.

**Materials and methods:**

A total of 48 patients, 28 males and 20 females, who were confirmed to have COVID-19 infection and received chest CT examination, were identified. The age range was 21–93 years old, with a mean of 54 ± 18 years. Of them, 33 patients received the first follow-up (F/U) scan, 29 patients received the second F/U scan, and 11 patients received the third F/U scan. The lesion region of interest (ROI) was manually outlined. A two-step registration method, first using the Affine alignment, followed by the non-rigid Demons algorithm, was developed to match the lung areas on the baseline and F/U images. The baseline lesion ROI was mapped to the F/U images using the obtained geometric transformation matrix, and the radiologist outlined the lesion ROI on F/U CT again.

**Results:**

The median (interquartile range) lesion volume (cm^3^) was 30.9 (83.1) at baseline CT exam, 18.3 (43.9) at first F/U, 7.6 (18.9) at second F/U, and 0.6 (19.1) at third F/U, which showed a significant trend of decrease with time. The two-step registration could significantly decrease the mean squared error (MSE) between baseline and F/U images with *p* < 0.001. The method could match the lung areas and the large vessels inside the lung. When using the mapped baseline ROIs as references, the second-look ROI drawing showed a significantly increased volume, *p* < 0.05, presumably due to the consideration of all the infected areas at baseline.

**Conclusion:**

The results suggest that the registration method can be applied to assist in the evaluation of longitudinal changes of COVID-19 lesions on chest CT.

## Introduction

The coronavirus disease 2019 (COVID-19) pandemic, caused by severe acute respiratory syndrome coronavirus 2 (SARS-CoV-2), poses a great threat to global public health (1). The number of people infected by COVID-19 is increasing rapidly all over the world. To date, more than 260 million confirmed cases have been reported, resulting in over five million deaths (1). The original strain of SARS-CoV-2 was identified at the end of 2019 in Wuhan, China. Since then, the virus has been evolving continuously, and several variants have emerged. As of 2022, five major variants of concern (VOC) have been reported, including Alpha (B.1.1.7), Beta (B.1.351), Gamma (P.1), Delta (B.1.617.2), and the latest Omicron (B.1.1.529) (2).

The earlier variants caused severe lung infections as a major symptom, which could be examined by imaging using X-ray and CT (3–6). Studies have reported that chest CT shows abnormal imaging features in nearly all the patients with COVID-19, thus it is a very sensitive diagnostic modality (7, 8). Therefore, from the time of the initial outbreak in late 2019 to early 2020, before the PCR test became widely available, CT was used as an alternative method for the diagnosis of COVID-19. The possible pathological mechanism in the lung infection is caused by diffuse alveolar damage and inflammatory exudation, which is similar to histologic findings seen in SARS-CoV-2 pneumonia (9, 10). Since CT has a high spatial resolution, it can be used to evaluate the morphology of lesions, the dynamic changes during the disease, and the response to treatment. The COVID-19 lesions were mainly located in the peripheral zone and close to the pleura, presenting as ground-glass opacity (GGO), consolidation, GGO mixed with consolidation, as well as vascular enlargement, interlobular septal thickening, and air bronchogram (11). The presence of GGO with single or multiple lesions suggests that the disease is in an early stage, while the bilateral multifocal consolidation can be seen in an advanced stage (12).

At the peak time of the global pandemics, many patients had severe symptoms and needed urgent medical care, resulting in high demand for hospital beds. Some patients died due to the lack of timely medical attention. To predict the need for medical resources, several studies have applied artificial intelligence (AI) algorithms to analyze the features of the COVID-19 lesions on CT. The results were combined with the clinical factors to predict the progression of patients that require more care, e.g., the need for mechanical ventilation and the admission to intensive care unit (ICU) (13–16). Although X-ray was not as sensitive as CT, it was also used to build models and achieved good results (17–20).

As the viruses evolved to Omicron, the disease has become milder, mainly affecting the upper respiratory system (21). The less severe symptoms of Omicron are in part due to the high rates of vaccination, which is known to be the key to the suppression of the viral load and the severe symptoms, e.g., pneumonia in the lung (22). However, patients are still dying from COVID-19 today. Lung pneumonia remains a major cause of death, and CT imaging still plays a key role in the hospital care of severe patients with COVID-19. Follow-up imaging can provide critical information related to the progression of the infection and the response to treatment.

Since CT can detect small areas of GGO (23), it is a promising imaging tool for longitudinal monitoring of the disease. Several studies have applied serial CT to assess the evolution of the COVID-19 lesions during the disease (24–26). Only qualitative evaluation was performed, e.g., by using a scoring system of 0–5 in each of 5 lobes in the lung, with a total score of 0–25. It was found that the peak lesion happened around 10 days after the onset of the infection (27). Segmentation methods may provide quantitative information (28). Although some AI-based segmentation tools have been developed (29–32), there are limited studies applying them in serial CT for follow-up evaluations (33). This may be due to the difficulty in the detection and precise segmentation of COVID-19 infection on CT at different stages, due to the high variation in texture, size, and position of infections on many CT slices. Visual evaluation of changes between two CT scans is subjective, and its validity may depend on the radiologists' experience, which is known to have a high variation (34).

To address these limitations and provide a solution, the purpose of this study is to develop a registration method between the baseline and follow-up (F/U) CT images, so the COVID-19 lesions can be compared in the co-registered lung areas. The method includes two steps, first using the Affine registration based on the body areas for alignment, and then followed by the nonrigid registration based on the segmented lung areas. Through the registration, the lesions at all the locations in the follow-up examinations can be objectively evaluated with the baseline lesions as references, to facilitate the quantitative volumetric comparison. The change in lesion volume at different follow-up times was reported, and the segmented volumes without and with the mapped baseline lesion as the reference were compared.

## Methods

### Subjects and computed tomography protocol

A total of 48 patients (28 males and 20 females) with COVID-19, who underwent chest CT in the radiology department of The First Affiliated Hospital of Wenzhou Medical University from 24 January to 3 March 2020, were enrolled in this retrospective study. The inclusion criteria were those who had positive SARS-CoV-2 nucleic acid in double swab tests (within 2 days, tested using real-time RT-PCR), without confirmation of another viral infection. The age range was 21–93 years old, with a mean of 54 ± 18 years.

The baseline CT was performed after the patient was admitted to the hospital, with the median [interquartile range (IQR)] of 5 (7) days after showing fever or other infection symptoms. Of them, 33 patients received the first F/U scan at the median (IQR) of 6 (6) days after the baseline CT. 29 patients received the second F/U scan at the median (IQR) of 5 (3) days after the first F/U CT, and 11 patients received the third F/U scan at the median (IQR) of 7 (2) days after the second F/U CT. All the patients in this study recovered and were discharged from the hospital.

This retrospective study was performed in accordance with the principles of the Declaration of Helsinki and was approved by the Ethics Committee in Clinical Research (ECCR) of the First Affiliated Hospital of Wenzhou Medical University (No. 2020008). The need for obtaining written informed consent from the patients was waived.

### Computed tomography protocol and COVID-19 lesion region of interest drawing

Non-contrast chest CT examinations were performed using a GE CT scanner (GE LightSpeed VCT 64-Slice, GE Healthcare, USA). The patients were scanned in the supine position during inspiratory breath-hold. The scanning range was from the apex to the base of the lungs. The parameters were as follows: tube voltage 120 kVp, tube current 50–350 mA, pitch 1.375 mm, matrix 512 × 512, and slice thickness 5 mm. Reconstruction was performed with a slice thickness of 1.25 mm, a lung window with a width of 1,500 Hounsfield units (HUs) and a level of −750 HU, and a mediastinal window with a width of 350 HU and a level of 40 HU.

All the 48 patients showed positive lesions on CT. The region of interest (ROI) of the pneumonia lesions was manually outlined. The drawing was done by three radiologists using the ImageJ software (https://imagej.nih.gov/ij/index.html) based on the consensus through discussion and cross-check. The outlined lesion areas included GGO, consolidation, or a mixture of GGO and consolidation. The radiologist carefully traced the boundary of all the infected areas on every CT slice containing the lesions inside both the lungs. Then, the segmented ROI results were examined by a senior radiologist with 9 years of experience interpreting chest CT for verification, and if needed, further modification was made. The total lesion volume in each patient was calculated by summing the outlined areas on all the slices.

### Lung segmentation and co-registration

The matching of the lung between two CT scans was completed in two steps. The first step was to apply the Affine registration using the whole-body area between the baseline images and follow-up images. The second step was to fine-tune the registration based on the segmented lung areas by using the non-rigid Demons registration algorithm.

The first task in this process was to perform the lung segmentation for each case. All the CT slices were combined to obtain a three-dimensional (3D) volume. Then, the middle slice in both the axial and coronal directions was determined. On the middle slices, a threshold of HU was set to identify the tissues (fat, muscle, heart, and bone) inside the body, and the remaining low HU regions were the lung areas. With the contour of the lung on the middle slices determined, the active contour algorithms were applied to segment the lung areas in the entire 3D volume. This technique, also called snakes, is an iterative region-growing image segmentation algorithm (35). Active contours can be defined as the process to obtain deformable models or structures with constraints and forces in an image for segmentation. The models describe the object boundaries or other features of the image to form a parametric curve or contour. Deformation is described by a collection of points that defines the contour of the image, by minimizing the energy function (35).

To complete the lung registration, the first step is to apply the Affine registration between the baseline images and follow-up images based on the entire body area. Then, the second step is to fine-tune the registration results on the segmented lung areas by using an intensity-based non-rigid registration, the Demons algorithm. The method applies a diffusion process to deform the lesion mask generated from the previous slice to the current slice, based on the distribution of intensities by iteratively minimizing the energy function, *E*, as shown below (36):


$$\begin{array}{l} E\left(u\right)=||F-M◦\left(T+u\right)|{|}^{2}+{\sigma_{n}}^{2}|F-M{|}^{2}||u|{|}^{2} \end{array}$$

where *M*, the moving image, is the segmented slice with the defined rectal mask that is to be deformed to segment *F*, the adjacent fixed image slice through an image transformation represented by the symbol °. For each iteration, the deformation field, *T*, is updated such that *T* = *T*+*u*, where *u* is the update factor and σ_*n*_ is the image noise ratio coefficient. Thus, the lung mask for the unsegmented slices is obtained by applying the correct transformation field to the mask of the moving image, M, on adjacent slices. The transformation field was found by solving for *u* by minimizing the energy function and given by (37):


$$\begin{array}{l} u=\frac{\left(M◦T-F\right)\nabla F}{\left[|\nabla F{|}^{2}+{\left(M◦T-F\right)}^{2}\right]} \end{array}$$

This process stopped when *u* was sufficiently small (*u* < 10^−3^).

For evaluating the registration performance, the corresponding mean squared error (MSE) of the lung areas was calculated as:


$$MSE=\;\frac{1}{N}\sum_{N}^{}{\left\|Moving\;Image-Referece\;Image\right\|}_{2}^{2}$$

where *N* is the total number of lung area voxels in one patient.

After registration, the lesion ROI on the baseline images was mapped to the follow-up images using the estimated geometric transformation matrix obtained from the registration, so the change can be visually compared. Then, by using the mapped baseline ROI as a reference, the radiologists performed the second-look ROI drawing on the F/U CT, and the measured lesion volumes were compared to those done in the first drawing.

### Statistical analysis

The descriptive statistics were presented. Since the COVID-19 lesion volume varied a lot among patients and was not normally distributed, the median and the interquartile range (IQR) were reported. For the change of the lesion volumes at F/U compared to its corresponding baseline value, the Wilcoxon signed-rank test was applied, and *p* < 0.05 was considered statistically significant. The registration quality after applying the Affine and the Affine + Demons algorithms were evaluated using the mean squared error (MSE) of the lung areas, compared using the Wilcoxon signed-rank test. The difference of the lesion ROI volume measured without and with the transformed baseline lesion as the reference was also compared using the Wilcoxon signed-rank test, with *p* < 0.05 as statistically significant.

## Results

### Change of the COVID-19 lesion volumes

**Figure 1:**
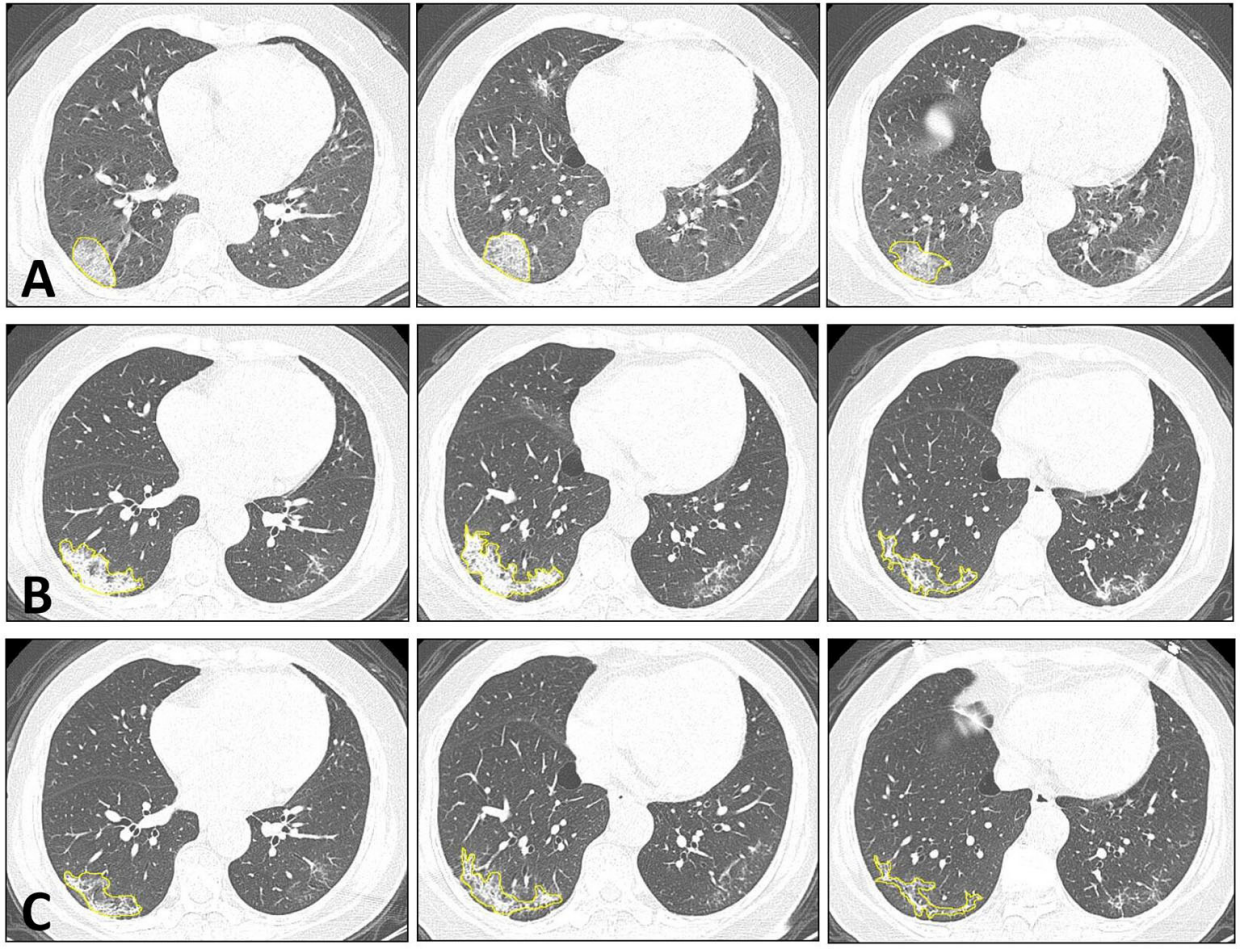
Dynamic changes of CT imaging in a 66-year-old female patient. At each time point, three selected CT slices are shown. **(A)** The baseline CT is taken approximately 3 days after the onset of infection. In the lateral and posterior basal segments of the right lower lung, increased ground-glass density is noted, which also exhibits the reversed halo sign and crazy-paving pattern in the lesion. **(B)** The first F/U is taken 12 days after the baseline CT. The lesion shows a higher density of consolidation change compared to the baseline. The overall infection area is increased, but the arc-shaped clearance near the pleura is noted, which indicates the beginning of the absorption phase. **(C)** The second F/U is taken 8 days after the first F/U. The lesion shows an obvious absorption, with smaller areas and a lower density.

**Figure 2:**
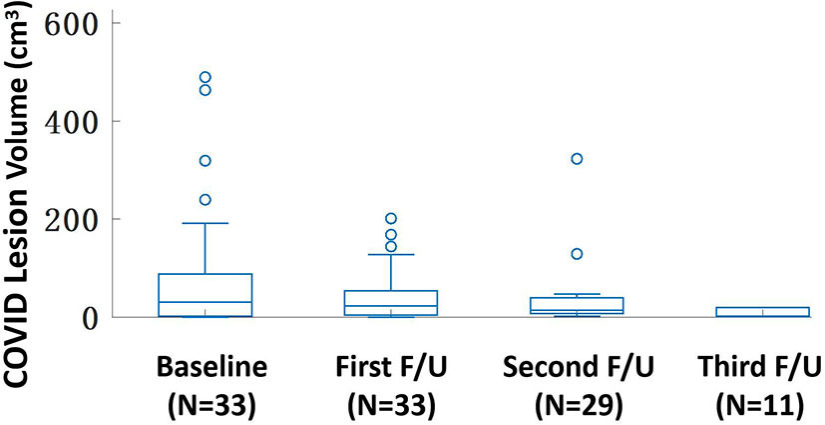
The box plot of the lesion volume distribution at the baseline and three follow-up CT examinations. The median (IQR) volume (cm^3^) is 30.9 (83.1) at baseline, 18.3 (43.9) at first F/U, 7.6 (18.9) at second F/U, and 0.6 (19.1) at third F/U. Only 11 patients have the third F/U, so the paired comparison is only done for the baseline, the first F/U, and the second F/U, all significantly different with *p* < 0.001.

Figure 1 shows one case example. The affected lesion area increased from the baseline to the first F/U, and then decreased at the second F/U scan. The total lesion volume in each patient was calculated, and the results from 33 patients who had the baseline and first F/U are shown in Figure 2. The median (IQR) volume (cm^3^) is 30.9 (83.1) at baseline, 18.3 (43.9) at first F/U, 7.6 (18.9) at second F/U, and 0.6 (19.1) at third F/U. The results of the other 15 patients who did not have F/U could not be compared, so they were not further analyzed. Overall, the volume is showing a significant decrease trend from baseline to the first F/U to the second F/U, with *p* < 0.001 for each paired comparison. But, depending on the timing of the CT, if the patient had the baseline scan during the early course of the disease, the volume might increase and then decrease, as shown in the case in Figure 1.

### Co-registration of the lung areas between follow-up and baseline

**Figure 3:**
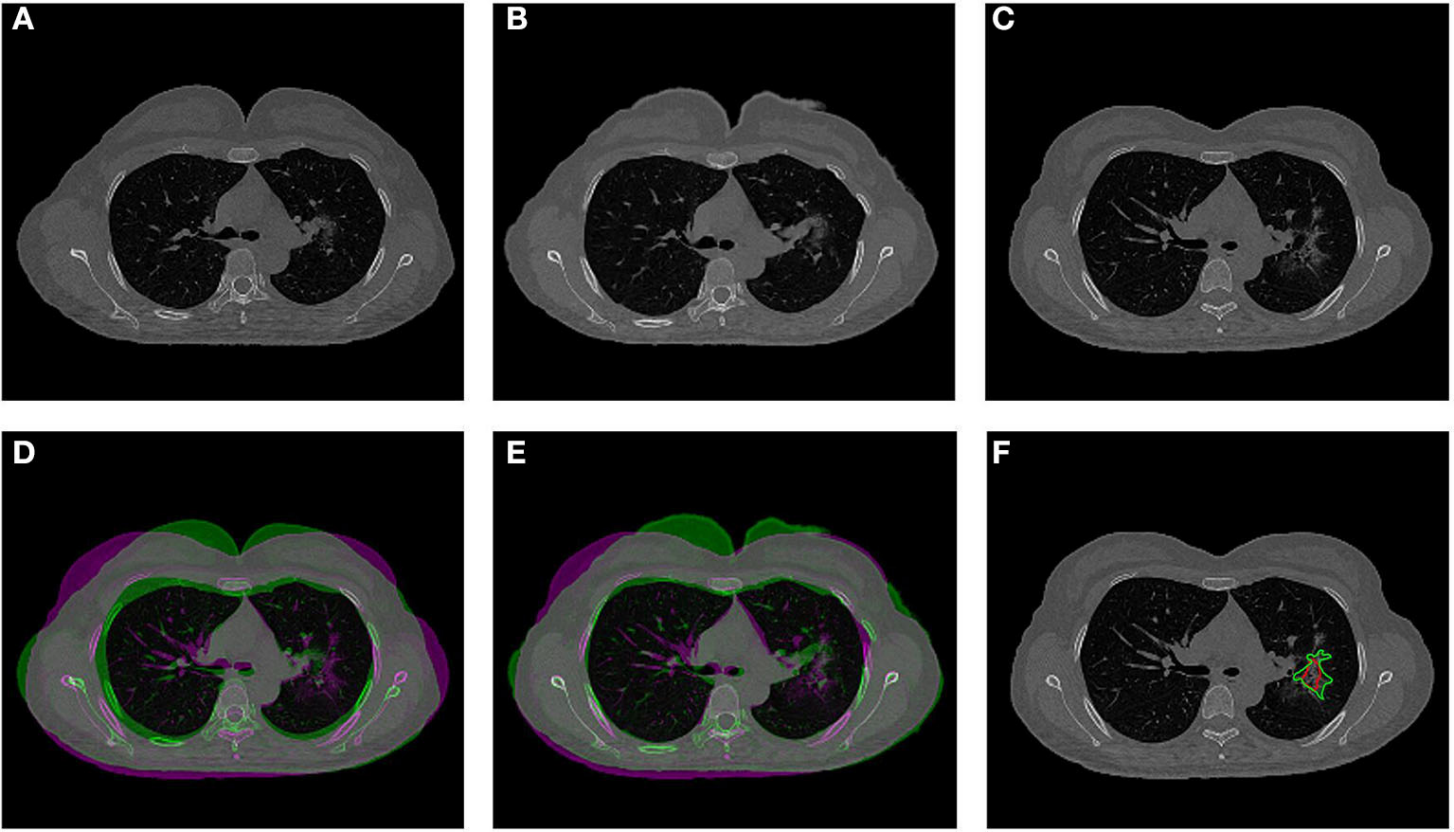
An example from a 52-year-old female patient. **(A)** Baseline image. **(B)** The transformed baseline image to match the F/U after completing the two-step Affine and Demons algorithms. **(C)** The first F/U image. **(D)** Comparison between the transformed baseline image after the first-step Affine registration and the first F/U image by the overlay. When the signal intensity on F/U is higher than on B/L, the pixel is labeled using purple color; when the intensity on F/U is lower than on B/L, it is labeled using green color. **(E)** Comparison between the final transformed baseline image and the first F/U image. It can be seen that the difference is smaller and the lung areas are better matched. **(F)** Overlay of the transformed B/L lesion (red contour) and the labeled first F/U lesion (green contour) on the F/U image, showing matching locations.

**Figure 4:**
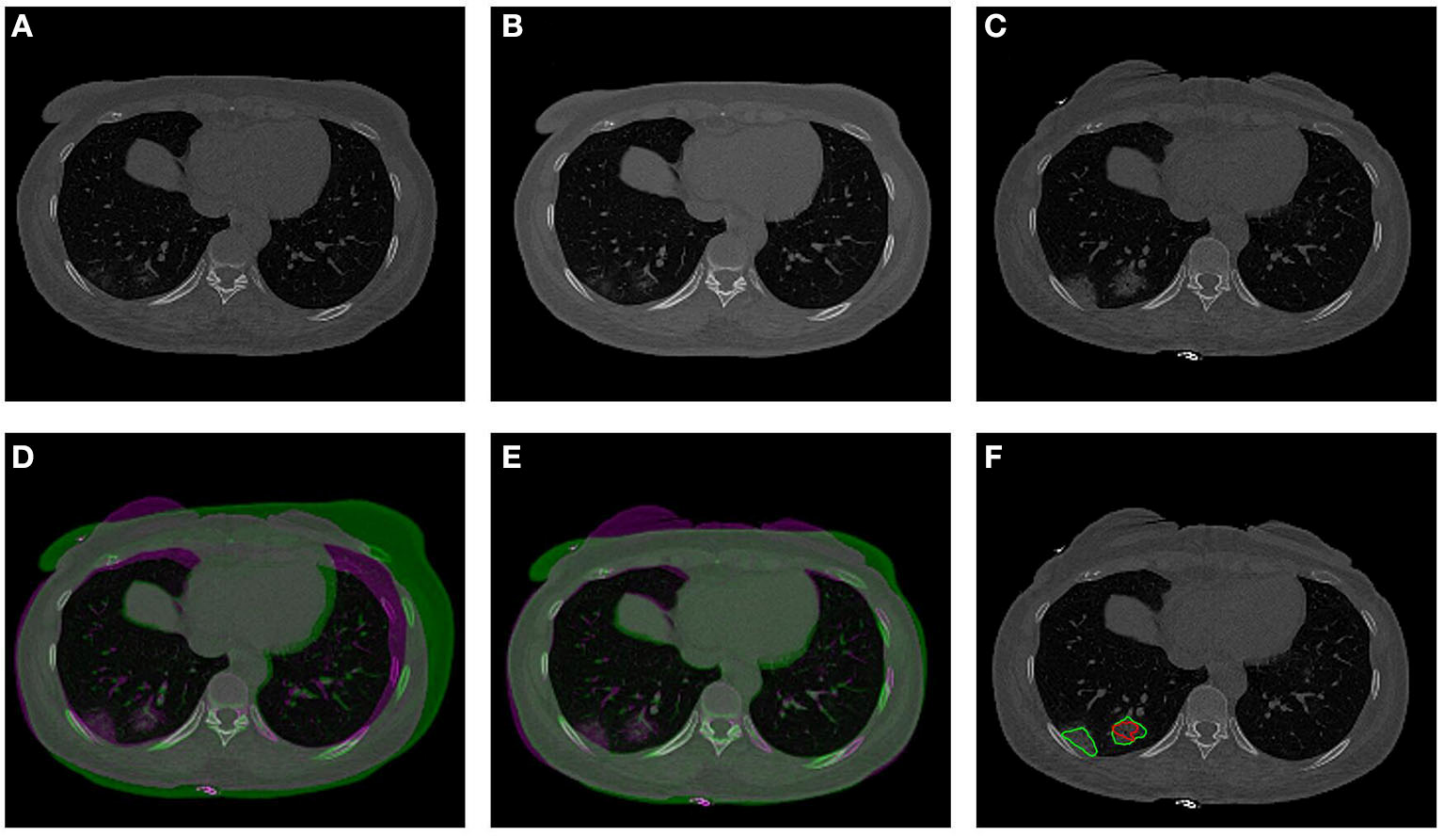
An example from a 57-year-old male patient. **(A)** Baseline image. **(B)** The transformed baseline image to match the F/U after completing the two-step Affine and Demons algorithms. **(C)** The first F/U image. **(D)** Comparison between the transformed baseline image after the first-step Affine registration and the first F/U image by the overlay. When the signal intensity on F/U is higher than on B/L, the pixel is labeled using purple color; when the intensity on F/U is lower than on B/L, it is labeled using green color. **(E)** Comparison between the final transformed baseline image and the follow-up image. It can be seen that the difference is smaller and the lung areas are better matched. **(F)** Overlay of the transformed B/L lesion (red contour) and the labeled first F/U lesion (green contour) on the F/U image. The size of the small lesion visible on the baseline has increased, and a new lesion close to the pleura is noted.

**Figure 5:**
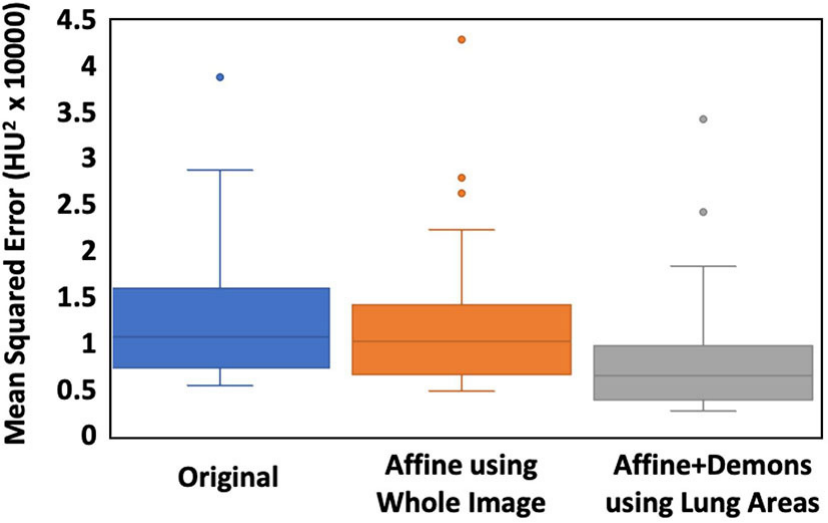
The box plot of the MSE between the baseline and the first F/U images. The first column is the difference calculated using the original images. The second column is calculated after the Affine alignment, and the third column is after the completed Affine + Demons registration. The MSE is decreased after the Affine, and further decreased after the Demons registration, and the difference is significant using the Wilcoxon signed-rank test (*p* < 0.001).

**Figure 6:**
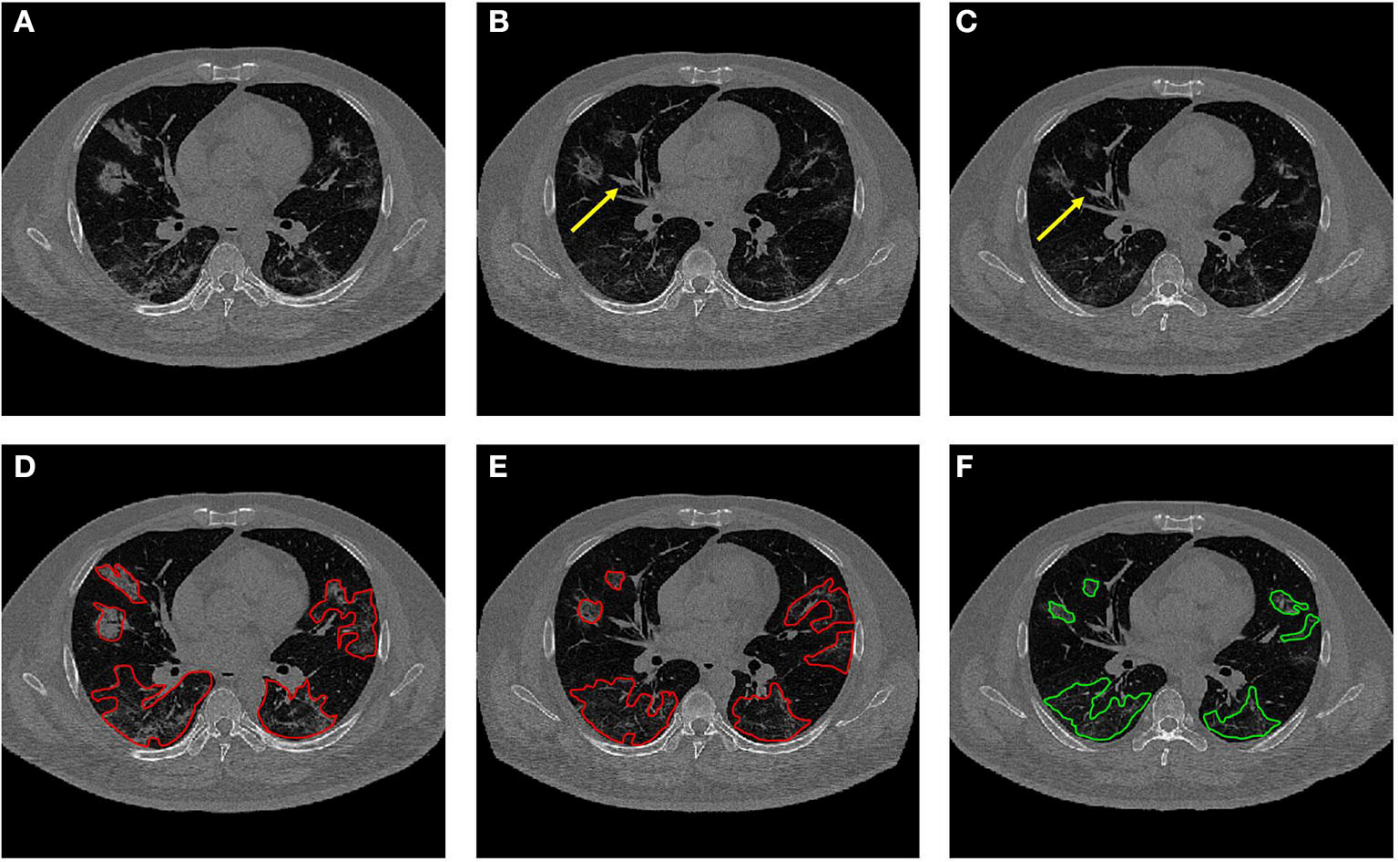
Dynamic changes of CT imaging in a 42-year-old male patient. **(A)** Baseline image. **(B)** The transformed baseline image to match the F/U after completing the two-step Affine and Demons algorithms. **(C)** The first F/U image. In **(B,C)**, it is noted that the vascular structures are better matched (yellow arrows). **(D)** The baseline CT with original lesion ROI. **(E)** The transformed CT with the mapped lesion ROI serves as the reference for the second-look ROI redrawing. **(F)** The original ROI on the first F/U CT.

The co-registration was applied to all the patients who received follow-up scans, using the MSE as the index for the evaluation of the registration quality. Figures 3, 4 show the co-registration results from two patients. Between the first F/U and baseline CT, the MSE calculated within the lung areas of 33 patients had a median value of 7,939 (IQR 5,841) after the Affine registration, which was decreased to 6,480 (IQR 5,821) after completing the second step of non-rigid registration using Demons algorithm. The box plot of MSE is shown in Figure 5, showing a significantly decreased MSE after completing the two-step Affine + Demons registration compared to using Affine registration (*p* < 0.001). The registration of baseline CT to the 2nd and 3rd F/U images also showed similar results with significantly decreased MSE. Most areas inside the lungs can be matched satisfactorily, as noted in the matching of the large vessels shown in Figure 6, suggesting that the proposed two-step registration method works satisfactorily.

After the registration was completed and the transformation matrix was obtained, the lesion contour drawn on the baseline images was mapped to the F/U images by using the transformation matrix obtained from the registration procedure. The mapped baseline lesion ROI is overlaid on the F/U images, so the lesions can be easily compared, as given in Figures 3, 4, 6.

### Region of interest redrawing using transformed baseline computed tomography as references

**Figure 7:**
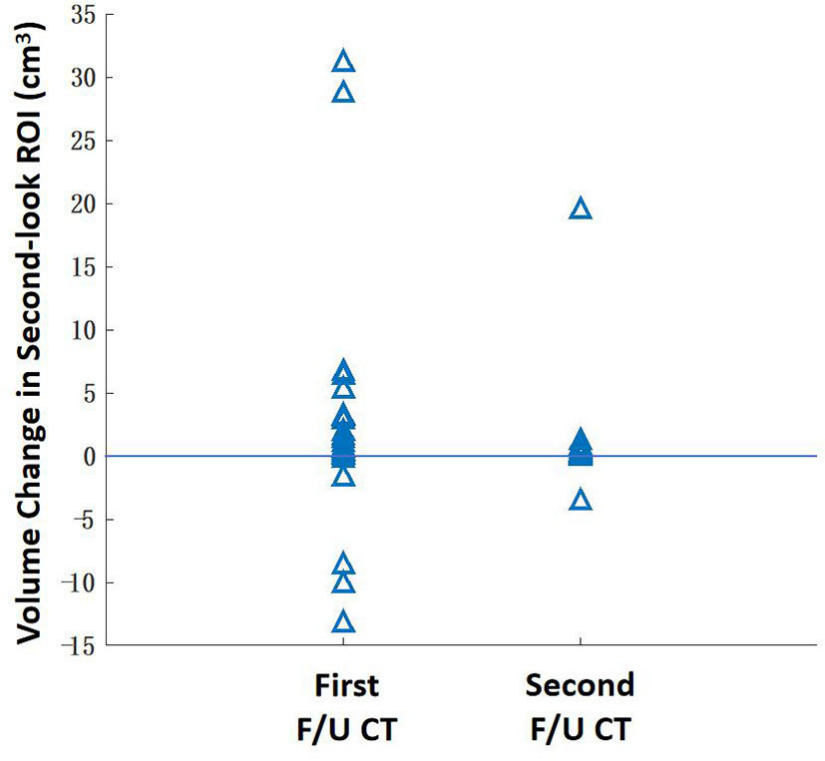
The change of the lesion ROI volume between the original ROI drawing and the second-look ROI redrawing using the mapped baseline ROI as the reference. The ROI of some patients with extensive diseases may show a higher volumetric change. Overall, the second-look ROI drawing has a significantly higher volume (*P* < 0.05) for the first F/U and second F/U CT.

After the registration was completed, the baseline ROI was mapped to the F/U images to serve as the reference for the second-look ROI redrawing. The volume in the second-look drawing was compared to the original drawing, and the difference is shown in Figure 7. The second-look ROI has a significantly higher volume, *p* < 0.05, by using the Wilcoxon signed-rank test.

## Discussion

In this study, we used a unique dataset of patients with COVID-19 receiving serial CT scans during hospitalization to perform the quantitative analysis of the lesion volume for evaluation of the dynamic changes. Depending on the timing of the initial CT from the onset of infection, some lesions may show regression at the first F/U, and some lesions may show progression first followed by regression at later times. All the patients in this study recovered and were discharged from the hospital. The chest CT features are known to be related to the course of the COVID-19 disease, and dynamic scans have been applied to monitor the patients' condition and response to treatment (11, 38). The typical COVID-19 pneumonia lesions present as the multiple ground-glass opacity (GGO), consolidation, and interstitial inflammation on chest CT (39, 40), which are consistent with our findings in this study.

Although several studies have applied CT to follow the change of the COVID-19 lung lesions, all of them are qualitative based on visual evaluation, which is subjective with a high variation. Second, although some AI-based detection and segmentation tools have been developed; they could not be applied to precisely segment the COVID-19 GGO lesions, due to the lack of clear tissue contrast. Therefore, these limitations motivated us to develop a registration method to match the lungs, so that the lesions on two CT scans could be directly compared. The images could then be further segmented to measure the volume of the COVID-19 lesions. We have shown that the two-step registration method using Affine + Demons algorithms could co-register the lung areas on the CT of the same patients taken at different times. The registration could significantly minimize the mean squared error. As demonstrated in the illustrated case examples, the transformed baseline images could match the lung areas and the large vessels inside the lung on the follow-up CT, which could provide more intuitive and objective views of the lesions for comparison. When using the transformed baseline images as references, the redrawn lesion ROI in the second-look ROI showed a significantly increased volume, which was likely due to the consideration of all the infected areas at baseline.

Coronavirus disease 2019 is a devastating disease that has spread all over the world. Although the gradual evolution to the much milder and more transmissible Omicron variant has drastically decreased the severe symptoms and the death rate, patients are dying from the disease today. Lung pneumonia remains a major complication that can lead to death, and CT is still the imaging modality commonly used for the management of hospitalized patients with COVID-19. During the initial outbreak in late 2019 to early 2020, CT played an important role. It was used to provide an alternative diagnostic method before the PCR tests became widely available and also used to detect suspicious infections for evaluation of the severity of pneumonia in the lung. Many studies have reported imaging findings related to COVID-19 infection in the lung, and there was an effort to develop a reporting system for diagnosis, named the COVID-19 Reporting and Data System (CO-RADS) (41), similar to those for the breast RADS (BI-RADS), prostate RADS (PI-RADS), and liver RADS (LI-RADS) cancers. The refinement and applicability of the CO-RADS is still a very active research area.

The longitudinal CT has been applied as a comprehensive, noninvasive imaging modality, which allows for the evaluation of lung parenchyma, patency of pulmonary and coronary arteries, and myocardial damage (42). In a study by Wang et al. (27) and Pan et al. (43), the volumetric changes in pulmonary involvement were analyzed based on the radiologist's reading. Due to the vague boundaries of the infected areas, the analysis was subjective and would be heavily influenced by the image quality and the radiologist's experience. Chen et al. pointed out the difficulty of using CT scans in asymptomatic SARS-CoV-2 infections (44). As the COVID-19 lesion was gradually absorbed in late F/U scans, the ROI delineation became challenging, and using the prior lesion as the reference would be helpful.

Although our registration method was developed and tested using serial CT of patients with COVID-19, it can be applied to all the patients presenting with lung diseases, including infection, inflammation, and pneumonia from all the etiologies, as well as for evaluation of benign lesions and primary and metastasis cancers. For the disease presenting as a solitary mass, it is easy to assess and compare, but for those presenting as multiple lung nodules and diffuse diseases, our registration method may provide a helpful tool for comparison.

There are some limitations to this study. First of all, the case number was small. The CT was collected during the initial outbreak in China when pneumonia was a major symptom that needed to be closely followed. At that time, a strict lockdown was imposed by the government to control the spread, and very soon the infection case dropped to zero. In this study, we only included patients who were healthy enough to receive serial CT scans, and all of them were recovered and discharged from the hospital. Patients who had severe COVID-19 or comorbid conditions that required mechanical ventilation support or admitted to ICU were not included. Second, it was difficult to determine the exact onset time of infection, so we reported the baseline CT time according to the sign of known first symptoms, which had a high variation. Third, the lesion ROI was manually drawn. Although some studies have tried to develop automatic quantitative analysis methods for the detection and segmentation of the COVID-19 infected lesions, the methods are not mature yet for routine use (29–32). Our ROI drawing was performed by several radiologists with consensus and cross-check, and the final results were verified by a senior radiologist. If large datasets are available for training and testing, more advanced algorithms developed using deep learning neural networks may provide novel techniques for lesion recognition and segmentation (45–49). Lastly, organ registration has become a mature technology, e.g., brain MRI of individual patients registered to a template for volumetric analysis of different structures (https://www.cortechs.ai/products/neuroquant/) and prostate MRI registered to ultrasound for guiding biopsies (https://koelis.com/fusion-guided-prostate-biopsy/). As long as the organ can be segmented, which can be done by deep learning, registration is straightforward, and the methods proposed in this study have the potential to be implemented for clinical use.

## Conclusion

In this study, we analyzed a dataset of patients with COVID-19 receiving serial CT, to gain more understanding of the evolution of the disease and the response to treatment. We developed a registration method using Affine + Demons algorithms to match the lung areas imaged by CT at different times, so the baseline lesion ROI could be mapped to the F/U images to serve as the reference for comparison. The method can allow intuitive and objective views of the lesions for comparison, and, thus, provide a standardization tool for monitoring the evolution of the disease and the response to treatment. The developed methods can be applied to many other lung diseases. Also, as some patients with COVID-19 may have sustained long-term damage to the lung, the method may provide an objective evaluation tool.

## Data availability statement

The original contributions presented in the study are included in the article/supplementary material, further inquiries can be directed to the corresponding author/s.

## Ethics statement

The studies involving human participants were reviewed and approved by the Ethics Committee in Clinical Research (ECCR) of the First Affiliated Hospital of Wenzhou Medical University. The Ethics Committee waived the requirement of written informed consent for participation. No potentially identifiable human images or data is presented in this manuscript.

## Author contributions

XC, YZ, and M-YS conceptualized and designed the study. GC, M-YS, and MW provided administrative support. XC, JZ, YL, and BC provided the study materials or patients. XC, JZ, and YL collected and assembled the data. XC, YZ, GF, and KN analyzed and interpreted the data. XC and YZ wrote the manuscript. XC, YZ, M-YS, JZ, YL, BC, KN, GF, MW, and GC gave the final approval of the manuscript. All authors contributed to the article and approved the submitted version.

## Funding

This study was supported by the Wenzhou Municipal Science and Technology Bureau, China (No. Y20180185), the Medical Health Science and Technology Project of Zhejiang Provincial Health Commission (No. 2019KY102), and the Key Laboratory of Intelligent Medical Imaging of Wenzhou (No. 2021HZSY0057, Wenzhou, Zhejiang, China).

## Conflict of interest

The authors declare that the research was conducted in the absence of any commercial or financial relationships that could be construed as a potential conflict of interest.

## Publisher's note

All claims expressed in this article are solely those of the authors and do not necessarily represent those of their affiliated organizations, or those of the publisher, the editors and the reviewers. Any product that may be evaluated in this article, or claim that may be made by its manufacturer, is not guaranteed or endorsed by the publisher.
